# Effects of acute substance use and pre-injury substance abuse on traumatic brain injury severity in adults admitted to a trauma centre

**DOI:** 10.1186/1752-2897-4-6

**Published:** 2010-05-26

**Authors:** Nada Andelic, Tone Jerstad, Solrun Sigurdardottir, Anne-Kristine Schanke, Leiv Sandvik, Cecilie Roe

**Affiliations:** 1 Department of Physical Medicine and Rehabilitation, Oslo University Hospital, Ulleval, Norway; 2 Department of Neuroradiology, Oslo University Hospital, Ulleval, Norway; 3 Sunnaas Rehabilitation Hospital, Nesoddtangen, Norway; 4 Centre for Clinical Research, Oslo University Hospital, Ulleval, Norway; 5 Faculty of Dentistry, University of Oslo, Norway; 6 Faculty of Medicine, University of Oslo, Norway

## Abstract

**Background:**

The aims of this study were to describe the occurrence of substance use at the time of injury and pre-injury substance abuse in patients with moderate-to-severe traumatic brain injury (TBI). Effects of acute substance use and pre-injury substance abuse on TBI severity were also investigated.

**Methods:**

A prospective study of 111 patients, aged 16-55 years, injured from May 2005 to May 2007 and hospitalised at the Trauma Referral Centre in Eastern Norway with acute TBI (Glasgow Coma Scale 3-12). Based on structural brain damages shown on a computed tomography (CT) scan, TBI severity was defined by modified Marshall classification as less severe (score <3) and more severe (score ≥3). Clinical definition of substance use (alcohol and/or other psychoactive substances) was applied when hospital admission records reflected blood alcohol levels or a positive drug screen, or when a physician verified influence by examining the patient. Pre-injury substance abuse (alcohol and drug problems) was screened by using the CAGE questionnaire.

**Results:**

Forty-seven percent of patients were positive for substance use on admission to hospital. Significant pre-injury substance abuse was reported by 26% of patients. Substance use at the time of injury was more frequent in the less severe group (p = 0.01). The frequency of pre-injury substance abuse was higher in the more severe group (30% vs. 23%). In a logistic regression model, acute substance use at time of injury tended to decrease the probability of more severe intracranial injury, but the effect was not statistically significant after adjusting for age, gender, education, cause of injury and substance abuse, OR = 0.39; 95% CI 0.11-1.35, p = 0.14. Patients with positive screens for pre-injury substance abuse (CAGE ≥2) were more likely to have more severe TBI in the adjusted regression analyses, OR = 4.05; 95% CI 1.10-15.64, p = 0.04.

**Conclusions:**

Acute **s**ubstance use was more frequent in patients with less severe TBI caused by low-energy events such as falls, violence and sport accidents. Pre-injury substance abuse increased the probability of more severe TBI caused by high-energy trauma such as motor vehicle accidents and falls from higher levels. Preventive efforts to reduce substance consumption and abuse in at-risk populations are needed.

## Background

Substance use (encompassing both alcohol and/or other psychoactive substances) is commonly associated with trauma [1,2]. The number of patients who have used substances while sustaining traumatic brain injury (TBI) is considerable, with an estimate of 36-51% showing some substance use on emergency admission to hospital [3,4].

Most studies related to substance consumption have focused on selected TBI populations such as victims of road traffic crashes [5], falls [6] or assaults [7]. In recent literature it has been debated whether the influence of alcohol increases [5] or decreases [8] the risk of more severe injuries, or if it has no effect [9]. The different views are primarily due to variations in the data collected, and a lack of consistency in methodology and outcomes. As reported by Parry-Jones et al. [4], most of the studies are conducted in the USA, which may limit applicability of findings to non-American countries, "given the potential influence of cultural factors on patterns of alcohol and drug consumption"[10].

Methods used to classify severity of head injury have included assessment of level of consciousness by the Glasgow Coma Scale (GCS) [11] or assessment of structural brain damage revealed on neuroimaging scans such as computed tomography (CT classification) [12]. The level of consciousness might be obscured in the acute phase due to substance use, in contrast to a more objective assessment of structural brain injury [13].

Several studies have assessed a link between substance use and clinical measures of TBI severity [3,4,14], but the data from Europe are limited [15]. However, there have been a few studies on the effects of substance use on anatomical brain injury based on CT classification [5,16]. A study by Cunningham et al. [5] reported that persons involved in motor vehicle accidents having tested positive for alcohol were approximately twice as likely to have more severe CT injuries than those who tested negative for alcohol. Ruff et al. [16] found that alcohol abuse before the injury, rather than alcohol intoxication levels at the time of injury, had a significant effect on the severity of intracranial injuries.

It is important to study the impact of substance consumption on TBI severity in different countries because of varieties in cultural acceptance of substances use, and also in order to identify significant abuse among TBI patients and identify those who might benefit from intervention. The present study is one of the few to date that have described the effects of substance use at the time of injury and pre-injury substance abuse on the level of anatomical brain injury severity shown on a CT head scan, across different causes of TBI.

The objectives of this study were:

1. To describe the occurrence of substance use at the time of injury in the moderate-to-severe TBI population admitted to the Trauma Referral Centre.

2. To detect patients at risk of having pre-injury substance misuse.

3. To determine whether substance consumption at the time of injury and pre-injury substance abuse affect the severity of TBI as measured by structural brain damage on the CT scan. On the basis of Cunningham's study [5], we hypothesised that patients who had consumed substances at time of injury (controlling for age, gender and cause of injury) would have CT evidence of more severe anatomical brain injury as compared to their non-influenced counterparts.

## Methods

This prospective study was part of a larger TBI project that comprises patients with acute TBI admitted to Oslo University Hospital, Ulleval, Norway during a period of 2 years, starting in May 2005. This hospital is the Trauma Referral Centre for the South-East region of Norway with a population of nearly 2.6 million (1.8 million in the East and 0.8 million in the South region). The definition of TBI and inclusion procedures have been described elsewhere [17,18]. Briefly, the study inclusion criteria were: (a) patients aged 16-55 years; (b) admitted with ICD-10 diagnoses S06.0-S06.9 within 24 hours of injury; considered as (c) moderate-to-severe TBI; (d) with known status of substance use at the time of injury (e) with CT scan of the brain performed within 24 hours post-injury; and (f) residing in East Norway. The initial severity of TBI was assessed by the GCS either at the time of emergency admission to the hospital or based on pre-intubation values assigned at the site of injury; GCS 3-8 represents severe and 9-12 moderate TBI [11]. In this study TBI severity was defined as structural brain damage shown on a CT scan using the Marshall classification, which is described more thoroughly below. We excluded patients with co-morbidities that might interfere with assessment of TBI consequences such as neurological disorders/injuries (n = 5) and known psychiatric diseases (n = 6). We omitted patients with previously diagnosed severe substance abuse disorders who were homeless or with unknown address (n = 14), and those being incarcerated (n = 4).

Over the inclusion period, 48 patients with moderate TBI and 99 patients with severe TBI who met the inclusion criteria were admitted to the hospital. Of these, 27 patients (12 in the moderate and 15 in severe TBI group) were not willing to participate in the study. A detailed comparison between participants and non-participants with moderate TBI showed no statistically significant differences in age, gender, GCS, cause of injury and substance use. In the participating group, a higher number had more severe intracranial pathology, but no statistically significant difference was revealed (p = 0.06). In the severe group there was no difference between participating and non-participating patients with regard to age, gender, substance use and intracranial pathology. A significantly higher number of participants had lower GCS (p = 0.02), and were injured in traffic accidents (p = 0.05).

Finally, we excluded four patients with missing CT and five with unknown substance use status on admission; thus, 111 patients were assessed in this study.

The study was approved by the Regional Committee for Medical Research Ethics, East Norway and the Norwegian Data Inspectorate.

## Measures

Baseline information including pre- and injury-related factors (e.g., socio-demographic and injury characteristics) was determined based on a systematic medical chart review and/or on data from the Trauma Register at Oslo University Hospital, Ulleval. The causes of injury were classified as follows: traffic accidents (irrespective of type), falls (irrespective of height) and others; assault and sport injuries were considered as subgroups of other causes. In this study, injury characteristics include both diagnostic and therapeutic variables and the functioning level at discharge from acute hospitalisation (as measured by Glasgow Outcome Scale, GOS) [19].

The Injury Severity Score (ISS) was used to indicated overall trauma severity [20]. The ISS is an anatomical scoring system that provides an overall score for patients with multiple injuries. Each injury is assigned to an Abbreviated Injury Scale (AIS) that classifies individual injuries by body regions on a 6-point ordinal severity scale [21]. The ISS scores range from 1 to 75 (best to worst) and are calculated by using the sum of the squares of the highest AIS scores in three different body regions. An ISS of 15 or greater is universally accepted as a definition of a major trauma patient. Trauma scores were extracted from the hospital's Trauma Register.

### Substance use

According to the definition of clinical judgment of substance use used by Bracken et al. [2], classifications were made when hospital admission records reflected blood alcohol levels or a positive drug screen, or when a physician verified influence by examining the patient, or when the patient reported recent substance ingestion. In this study we used dichotomous classification for substance use: yes/no. We decided to use the clinical definition of substance use to enhance the utility of physician observations "which reflect concern that different substances not detected on routine laboratory testing may indeed have influenced the patients, and that physicians are required to treat the patients before laboratory results are available" [2].

However, many patients are routinely tested for alcohol ingestion during clinical TBI assessment on emergency admission to the hospital (enzymatic method). The toxicology screening for other substances is done on clinical indications (immunological screening method in urine). If present, blood alcohol concentrations (BAC) as well as screening of the other substances in urine analyses were derived from admission laboratory files.

### Pre-injury substance abuse

We used the CAGE questionnaire (**C**ut down, **A**nnoyed, **G**uilty, **E**ye-opener) as a standard patients interview for screening pre-injury substance abuse in our TBI population [22]. The CAGE consists of four questions that address the lifetime drinking experience. Questions are also modified to address drug use experience. The CAGE is popular in clinical settings because of its brief administration time [23]. Previous studies have shown that the CAGE may be a useful screening test for substance abuse in the TBI population [24]. A score of 2 or more is considered a cut-off score indicating clinically significant alcohol and/or drug problems [23]. The CAGE interviews were administrated as part of a follow-up study and were available for 88 patients.

### Structural brain damage (CT)

TBI severity was measured by the structural brain damage shown on head CT scan. Patients underwent a CT head scan shortly after admission. A second CT was obtained within 6-24 hours after injury. Findings from the first and second CT scans were categorised according to diagnostic categories of types of anatomical abnormalities as classified by Marshall et al. [12]. A neuroradiologist (the second author) reviewed the CT findings. Scores from the "worst" CT were used in the final analyses [25]. The original Marshall classification ranges from 1 to 4, with separate categories for any lesion that is surgically evacuated and non-evacuated mass lesions. Few patients were observed in category 4 and in separate categories (Table 1), thus precluding analyses in all the Marshall categories. Therefore, the original Marshall classification was subdivided into two groups [5]. The first group included patients with Marshall score <3 (less severe brain injury) and the second group included those with Marshall score ≥3 (more severe brain injury with significant intracranial abnormalities).

**Table 1:** Distribution of "worst" CT scan findings assigned by Marshall score (N = 111).

Marshall score	n (%)
1 No visible intracranial pathology	14 (13)
2 Cisterns present with midline shift 0-5 mm; no high or mixed density lesion > 25 ml.	36 (32)
3 Cisterns compressed or absent with midline shift 0-5 mm.	43 (39)
4 Midline shift > 5 mm;or surgically evacuated lesion;and non-evacuated high or mixed density lesion >25 ml	18 (16)

### Statistical analysis

Descriptive data are presented using the proportions and mean values with standard deviations (SD), or the median with interquartile range (the 25^th ^and 75^th ^percentile values). The Mann Whitney U-test was used to compare differences between participants and non-participants, and when analysing differences between modified Marshall groups regarding gender and length of Intensive Care Unit (ICU) and acute hospital stay. T-tests were used when analysing differences in age, GCS, ISS and BAC levels. Further, the Chi-square test with contingency tables was applied when studying associations between categorical independent variables.

Logistic regression analyses was used to evaluate effects of substance use at the time of injury and pre-injury substance abuse on TBI severity, and odds ratios (OR) with confidence intervals (95% CI) were calculated. Substance use and pre-injury substance abuse were entered as predictor variables and analysed separately (crude OR) against the Marshall groups, which comprised the dependent variable. Possible confounding variables studied in the multivariate regression analysis (adjusted OR) were gender and age, as well as education levels and the cause of injury (as these differed significantly in the two severity groups). The final regression analysis was also adjusted for substance use and pre-injury substance abuse. Age was recorded in four categories (in 10-year intervals) and cause of injury was dichotomised into traffic accidents and others. The categories with highest number of patients were reference groups. For the categories substance use and CAGE, the reference group consisted of patients who screened negative for substance use and abuse. All statistical tests were two-sided and the 5% significance level was used. Statistical analyses were performed using SPSS for Windows, version 14 (SPSS Inc, Chicago, IL).

## Results

### Demographic and injury characteristics

Table 2 shows the main demographic and injury characteristics of all study patients (n = 111) in relation to anatomical severity of TBI as measured by the modified Marshall classification (score <3 less severe, score ≥3 more severe TBI). Fifty-five percent of the patients (n = 61) had severe anatomical injuries on initial CT scan. There was no statistically significant age difference (p = 0.69) between the two severity groups, while the gender difference approached significance (p = 0.08). Education was significantly lower in the group with more severe injuries (p = 0.002). Furthermore, no significant differences were found in marital and employment status (p = 0.54 and p = 0.40, respectively). Two-thirds of the patients in the group with more severe injuries were involved in traffic accidents. The GCS and ISS scores differed significantly between the severity groups, as did several other injury-related variables including in-hospital mortality (21% vs. 0%), the length of acute hospital stay and the global functioning at discharge from acute hospitalisation (see Table 2).

**Table 2:** Demographic and injury characteristics in relation to Marshall groups (score <3 less severe, score ≥3 more severe TBI).

Variables	Less severe TBI (n = 50) (%)	More severe TBI (n = 61) (%)	p-value	Total (N = 111) (%)
**Gender**			0.08	
Male	43 (86)	44 (72)		87 (78)
Female	7 (14)	17 (28)		24 (22)
**Age (years)**			0.69	
Mean ± SD	31.7 ± 10.7	32.6 ± 12.4		32.2 ± 11.6
**Education**			0.002	
0-9 years	11 (22)	4 (6)		15 (14)
10-12 years	14 (28)	33 (54)		47 (42)
≥ 13 years	25 (50)	18 (30)		43 (39)
Missing	0 (0)	6 (10)		6 (5)
**Marital status**			0.54	
Married/live with	27 (54)	35 (59)		62 (56)
Divorced	4 (8)	7 (12)		11 (10)
Live alone	19 (38)	17 (29)		36 (32)
Missing	0 (0)	2 (3)		2 (2)
**Employment**			0.40	
Employed	39 (78)	46 (81)		85 (77)
Unemployed	3 (6)	6 (11)		9 (8)
Retired/Disabled	8 (16)	5 (9)		13 (12)
Missing	0 (0)	4 (7)		4 (2)
**Cause of injury**			0.04	
Traffic accidents	23 (46)	42 (69)		65 (59)
Falls	17 (34)	11 (18)		28 (25)
Others	10 (20)	8 (13)		18 (16)
**Substance use**			0.01	
No	20 (40)	39 (64)		59 (53)
Yes	30 (60)	22 (36)		52 (47)
**Glasgow coma scale (GCS)**			0.001	
Mean ± SD	8.9 ± 2.9	5.5 ± 2.5		7.0 ± 3.2
**Injury severity score (ISS)**			0.001	
Mean ± SD	25.2 ± 14.6	35.0 ± 10.7		30.6 ± 13.5
**Type of injury**			0.67	
Isolated TBI	20 (40)	22 (36)		42 (38)
Multiple traumas (incl. TBI)	30 (60)	39 (64)		69 (62)
**Intracranial surgery**			0.001	
No	39 (78)	21 (34)		60 (54)
Yes	11 (22)	40 (66)		51 (46)
**Medical complications**			0.001	
No	35 (70)	12 (20)		47 (42)
Yes	15 (30)	49 (80)		64 (58)
**ICU length of stay (days)**			0.001	
Median (IQR)	1.5 (6)	12 (14)		5 (13)
**Length of acute hospital stay (days)**			0.001	
Median (IQR)	5 (6)	12 (12)		8 (11)
**Glasgow outcome scale (GOS)at discharge**			0.001	
1 (dead)	0 (0)	13 (21)		13 (12)
2 (vegetative state)	0 (0)	14 (23)		14 (13)
3 (severe disability)	19 (38)	28 (46)		47 (42)
4 (moderate disability)	31 (62)	6 (10)		37 (33)
5 (good recovery)	0 (0)	0 (0)		0 (0)

ICU = Intensive Care Unit, IQR = interquartile range

### Substance use

**Figure 1:**
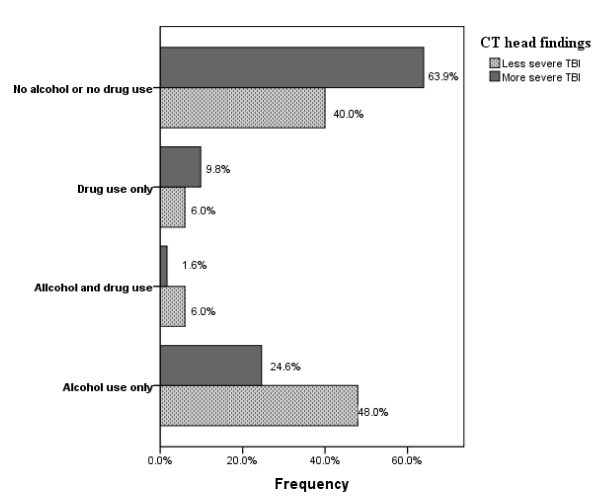
**Frequency of substance use by modified Marshall classification into less severe (score <3) and more severe TBI (score ≥3)**.

Forty-seven percent of all the patients used some kind of substances at the time of injury; alcohol ingestion was found in 35%, influence by other substances in 8%, and combined consumption in 4% of patients. Figure 1 shows the frequency of substance use in the severity groups (using Marshall Classification).

Seventy-five percent of results regarding substance use were derived from blood or urine analyses and 25% by clinical judgment. There was no statistically significant difference regarding severity of intracranial injuries between tested and non-tested patients in the group considered as positive for substance use (χ^2 ^= 1.34, p = 0.25). BAC levels were available in 31 of 39 patients considered as alcohol-influenced. In 87% of these, the BAC was >100 mg/g; with mean BAC values of 185 mg/g in the less severe group and 210 mg/g in the more severe group, p = 0.44). Of eight patients who were not BAC tested, six were in the more severe TBI group. Of six patients tested for use of other substances, four were in the more severe group. Of seven patients who self-reported drug use at the time of injury, three were in the severe TBI group. One of four patients with combined alcohol and drug use was in the more severe TBI group. Cannabis was the most commonly detected substance (54%) followed by benzodiazepines (46%), amphetamine (31%), barbiturates, cocaine and LSD (24%) and methadone (8%).

Only 12% of the females were in the positive substance use group. The mean age was similar in both the substance-positive and substance-negative groups [31.8 years (SD 11.5) vs. 32.6 years (SD 11.8), p = 0.70]. Sixty percent of substance positive patients were under 35 years of age. Alcohol ingestion was strongly related to the cause of injury; 29% of patients injured in traffic accidents were in the positive group, in contrast to 71% of those injured in falls and 92% of assault patients (χ^2 ^= 25.01, p = 0.001). There were no differences in the mean GCS scores between substance-positive and negative groups [6.9 (SD 3.2) vs. 7.2 (SD 3.1) respectively, p = 0.72]. Patients in the substance-negative group had higher mean ISS than those in the positive group with a statistically significant difference [33.3 (SD 13.9) vs. 27.4 (SD12.3), p = 0.02]. Substance use was significantly higher in the less severe Marshall group (60% vs. 36% p = 0.01). All 13 patients who deceased during the acute hospital stay suffered from severe TBI; only two of these were influenced by alcohol and one by other substances at hospital admission.

The median length of acute hospital stay differed significantly between substance-positive and substance-negative groups (6 days, interquartile range 11 vs. 8 days, interquartile range 11, p = 0.001). The global functioning at hospital discharge was better in the substance-positive vs. substance-negative group (GOS level 4: 39% vs. 29%).

### Pre-injury substance abuse

CAGE data were available in 80% (n = 88) of all the patients. Of the remaining 23 patients, 13 were deceased as mentioned above; eight were not able to participate in the interview because of communication disorders and two dropped out. Positive screening for pre-injury substance problems (CAGE cut-off ≥2) was found in 26% of patients (n = 23). Of these, 13 were influenced by alcohol on admission, five by other substances and two by poly-substances (χ^2 ^= 20.4, p = 0.001). Only 3 females were in the CAGE risk group. The mean age in the CAGE positive group was 33.6 years (SD 12.1) vs. 30.1 years (SD 11.1) in the negative CAGE group (p = 0.22). Eleven patients in the risk group were injured in traffic accidents, nine in falls and three in assaults. Eighty-four percent of patients with lower education level in the age group younger than 35 years of age were at risk of having significant pre-injury substance abuse, as well as 60% of the patients above 35 years with higher education level (≥13 years).

Table 3 shows injury characteristics (diagnostic, therapeutic and functioning variables) in substance abuse vs. no substance abuse groups.

**Table 3:** Injury characteristics and therapeutic variables in substance abuse versus no substance abuse groups.

Variables	Substance abuse (CAGE ≥2) n = 23	No substance abuse (CAGE <2) n = 65
Glasgow coma scale (GCS)		
Mean ± SD	7.7 ± 3.2	7.7 ± 3.1
Injury severity sore (ISS)		
Mean ± SD	30.1 ± 12.9	28.3 ± 13.0
Intracranial surgery		
Rate	52%	35%
Intracranial pressure (ICP)		
Measurement rate	57%	47%
Artificial ventilation		
Rate	81%	68%
Tracheostomy		
Rate	57%	39%
Artificial ventilation days		
Median (IQR)	12 (19)	2 (15)
Medical complications >1		
Rate	30%	15%
ICU length of stay		
Median (IQR)	9 (15)	4.5 (12)
Length of acute hospital stay		
Median (IQR)	9 (12)	7.5 (10)
Glasgow outcome scale (GOS)		
Level ≥4	30%	45%

ICU = Intensive Care Unit; IQR = interquartile range

The mean GCS was similar in both CAGE groups. The mean ISS was not found to be significantly higher in patients with a positive CAGE screen. However, the proportion of patients with positive CAGE was found to be higher in the more severe group as compared to the less severe group (30% vs. 23%, p = 0.45). Of 13 CAGE positive patients who were influenced by alcohol on hospital admission, six were in the more severe Marshall group, as well as four of those who were influenced by other substances. Intracranial surgery was performed with half of the CAGE positive patients. At discharge from the acute hospital stay, severe disability was shown in two-thirds of patients in the substance abuse group.

The unadjusted and adjusted effects of acute substance use and pre-injury substance abuse on the unfavourable intracranial severity group (Marshall ≥3) are shown in Table 4. Binary logistic regression analyses included the 88 patiens with available CAGE data. In both unadjusted and adjusted models, substance use on admission tend to decrease the probability of more severe intracranial injuries (OR 0.52; 95% CI 0.23-1.24, p = 0.14 and OR 0.39; 95% CI 0.11-1.35, p = 0.13, respectively). In the regression analyses adjusted for age, gender, education, substance use at time of injury and cause of injury, pre-injury substance abuse (CAGE ≥2) significantly increased the probability of more severe TBI (OR 4.05; 95% CI 1.05-15.64, p = 0.04). According to covariate analysed female gender, age group 46-55 years, lower education level and traffic accidents tend to increase the probability of more severe TBI (data not shown). The final adjusted regression model predicted more severe TBI in 70% of cases (see Table 4).

**Table 4:** Risk for severe TBI (Marshal ≥3) associated with substance use at time of injury and pre-injury substance abuse (n = 88).

	Number of cases n (%)	Less severe TBI n (%)	More severe TBI n (%)	OR (95% CI) Unadjusted	OR (95% CI) Adjusted for age and gender	OR (95% CI) Adjusted for age, gender, education, cause of injury, subst. use/CAGE
Substance use						
No	43 (49)	20 (42)	23 (58)	1*	1*	1*
Yes	45 (51)	28 (58)	17 (42)	0.52 (0.23-1.24)	0.60 (0.24-1.53)	0.39 (0.11-1.35)
				p = 0.14	p = 0.28	p = 0.14

CAGE						
< 2	65 (74)	37 (77)	28 (70)	1*	1*	1*
≥ 2	23 (26)	11 (23)	12 (30)	1.44 (0.56-3.74)	1.66 (0.60-4.61)	4.05 (1.05-15.64)
				p = 0.45	p = 0.32	p = 0.04

The data are presented as OR (odds ratio). *Reference category. OR greater than 1 increases probability of severe TBI. OR less than 1 decreases the probability of severe TBI.

## Discussion

### Demographic and injury characteristics

Demographic and injury characteristics in the present study of a TBI cohort aged 16-55 years admitted to the Trauma Referral Centre with acute TBI are comparable to those of other TBI populations [8,13]. All subjects from East Norway with moderate-to-severe TBI (GCS 3-12) in need for acute neurosurgical check-up and care are referred to this Trauma Referral Centre. Participants in this study were representative of their cohort; the refusal rate was about 20% as in existing literature [26], and the substance use at the time of injury did not differ between participants and non-participants.

The level of consciousness might be obscured in acute settings due to substance use at the time of injury, medical sedation or paralysis [13]. In this study, the mean GCS score did not differ significantly between the substance-positive and substance-negative groups of patients, agreeing with results reported by Sperry [27]. In contrast, assessment of structural brain damage by neuroimaging is not influenced by state of consciousness. Therefore, we defined the severity of TBI in this study by structural brain damage as shown on CT scans. There is evidence that the CT scan can assist in discriminating less severe from more severe TBI using the Marshall scores as a standard measure of anatomical classification of severity [5]. Fifty-five percent of patients in this study had injuries that could be categorised as more severe.

### Substance use

In this study, almost half of the patients showed substance use upon admission to the hospital. The proportion of patients found to be under the influence of alcohol was 35%, which is higher than in previous Norwegian studies [28,29]. A trend towards increasing alcohol consumption in the general Norwegian population during the last decades [30] as a result of the increased number of regular drinkers [31] as well as higher consumption in Oslo/Eastern Norway than in other regions may explain this result [32]. Norway is, however, in the lower range of international statistics on alcohol consumption as compared to other Western countries [32]. This could be explained by limitation in availability of alcohol, as well as the high taxation on alcoholic beverages in Norway. The alcohol consumption rate shown in this study was within the range of those reported in a recent review of TBI epidemiology in Europe (24-51%) [15] and other international studies [3,4]. This study also confirms that alcohol is the most common substance used in the TBI population [4,8].

The use of other psychoactive substances was found in 12% of the total TBI sample. This rate is lower than those presented in international studies [14], and is biased by clinical routines on admission and the clinical definition of substance use. In agreement with studies on illicit drug use in TBI populations [14], cannabis was the most frequently detected drug. It is well known that cannabis is the most frequently used illegal drug in the general population in Norway [33]. The proportion of patients that use substances while driving motor vehicles was more than five times higher in the present study than in a recent study on the prevalence of alcohol and illicit drugs among motor vehicle drivers (aged ≤ 54 years) in South-Eastern Norway [33]. Our findings may indicate that persons who sustain TBI are not representative of the general population. However, a considerably lower proportion of traffic injury cases in this study were alcohol-influenced than in older TBI literature [14]. This may represent the reduction of the legal limit for BAC from 0.5 to 0.2 mg/g in 2001 [33], effectiveness of campaigns and programmes for reducing drinking and driving, and a decrease in the incidence of traffic-related TBI during the last decades [17].

The proportion of patients with substance use was significantly higher in the less severe Marshall group. Falls and other injuries were the main causes of injury in this severity group. The percentage of patients under the influence of alcohol on admission and injured by falls or assaulted was in agreement with other Nordic studies [9,29]. Northern European countries are often characterised as nations with infrequent but heavy episodes of drinking ("dry" culture), with higher tolerance toward excessive drinking, and higher frequencies of alcohol-related injuries than Southern European countries where alcohol use is more integrated into everyday life ("wet" culture) [34].

As hypothesised previously, we expected that substance use on admission could have a potentiating effect on anatomical brain injury, as measured by findings on the "worst" head CT scan within 24 hours of injury [5]. Contrary to expectation, substance use tended to decrease the probability of more severe intracranial injury in the adjusted logistic regression (OR = 0.39). However, strong conclusion should not be drawn due to insufficient statistical power in the analysis (power 0.58, alpha 0.05) [35]. Cunningham's results [5] showing that alcohol potentiated the severity of TBI among victims of motor vehicle crashes were not replicated in this study.

It has been reported that acute alcohol intoxication exerts both detrimental and beneficial effects on the injury severity and outcome of TBI, although mechanisms have not been determined. The hypothesis that alcohol-inebriated victims injured by falls on stairs sustain more severe intracranial injuries because of delayed reaction time and inadequate protection reflexes was not supported [6]. Smink et al. [36] could not demonstrate a relationship between alcohol concentration and the severity of traffic accidents. Furthermore, alcohol and methamphetamine use was found to be associated with decreased mortality in a retrospective study of severe TBI [8]. Ruff et al. [16] also found that alcohol intoxication at the time of injury "was not associated with poor outcome in those who survived long enough to reach hospital". Based on experimental research, Kelly [37] reported that acute alcohol intoxication may have neuroprotective effects in traumatic brain injuries as a result of "ethanol-induced inhibition of N-methyl-D aspartate receptor-mediated (NMDA) excitotoxicity". Dose-dependent effects are also reported, with a better outcome in animals obtained with low and moderate ethanol doses as compared to no- and high-ethanol groups [38]. According to Tien et al. [39], low to moderate BAC may be beneficial in patients with severe brain injury from blunt head trauma, while high BAC seems to have a deleterious effect on in-hospital death in these patients. Although the focus of our study was not to assess whether BAC levels impact the severity of structural brain injury, the mean BAC level was found to be similar in both severity groups.

The considerable number of patients with substance use in the present study shows that the use of alcohol and drugs is a major risk factors for TBI [3,9,39]. Health-related and psycho-social consequences of TBI, as well as the economical burden of these injuries underline the importance of preventive efforts targeting at-risk populations.

### Pre-injury substance misuse

Screening by the CAGE questionnaire showed that 26% of patients reported pre-injury substance abuse (problems with alcohol and/or other substances). Around two-thirds of these patients misused alcohol. This study shows a lower proportion of pre-injury substance misuse than in TBI literature (40-55%) reviewed from 1994-2004 [4]. This rate is also slightly lower than that reported in a recent study on a TBI population from Australia (21% alcohol and 9% drug dependence) [10]. In our study, we omitted patients with pre-existing substance abuse disorders, which could explain these lower rates. Lower sensitivity of the CAGE-drug questionnaire as compared to the CAGE-alcohol questionnaire [24] is a methodological limitation, as the cut-off score used. If we include patients with a CAGE score of one (8 patients, 9%), those omitted due to pre-diagnosed severe substance abuse disorders (14 patients, 14%) and the pre-injury substance misuse group (26%), a total proportion of 49% reached the range of estimates presented in the literature [4,10]. We based our findings on self-reports from the patients (the CAGE interview), thus possibly under-reporting illicit drug use, since patients may be unwilling to report engagement in illegal activities [4]. Prior history of substance abuse is less often found in persons treated in Trauma Referral Centres than in rehabilitation populations [3]. However, the occurrence rate of pre-injury substance abuse in this study is higher than in a Danish study [40], where 5.8% of the intracranial lesions group were diagnosed with substance misuse by the ICD-9 criteria. In our study, 26% of all patients with ICD-10 diagnoses of intracranial lesions had a positive CAGE screening for pre-injury substance problems.

CAGE positivity is usually equivalent to being a heavy drinker [41]. When we extracted results of patients with positive CAGE-alcohol, the proportion shown was more than two times higher than the rate of heavy drinkers found in a Norwegian survey in 1998 [30]. One population at risk of pre-injury substance misuse in this study was a male, younger than 35 years of age, with a lower education level (≤12 years). The other population was single males, older than 35 years, with a high school education. It has previously been reported that drinking serves an important social function for young people [10], and that increased consumption of alcohol is found in subjects with a higher level of education and higher income [30,32].

We found in the adjusted regression analysis that patients with pre-injury substance problems showed significantly increased probability of more severe TBI as compared to their counterparts (OR 4.05). Chronic alcohol use has consistently been found to be associated with more severe traumatic brain injuries. Ronty et al. [42] reported that pre-injury alcohol abuse precipitated the development of more severe structural traumatic brain damage on CT examinations. A strong association between history of excessive alcohol use and poor outcome for all types of intracranial diagnoses and greater prevalence of mass lesion was reported by Ruff at al. [16]. Several other studies have reported an association between alcohol abuse and greater severity of TBI as measured by posttraumatic amnesia, neuropsychological tests and global functioning [3,4,14]. According to previous experimental studies, chronic alcohol exposure may exacerbate TBI, probably as the "effect of imbalance of up-regulation of NMDA receptor activity and down-regulation of gamma-aminobutyric acid (GABA) receptor function resulting in excitotoxicity" [37].

The association shown in this study between pre-injury substance abuse and more severe intracranial injuries underlines the importance of identifying persons at risk who would benefit from intervention related to substance abuse. A routine alcohol and psychoactive substance screening of TBI patients at the emergency admission may pinpoint this population [1].

In the adjusted regression analyses, female gender tended to have increased probability of more severe intracranial injury, in agreement with a previous study on females under 50 years of age [43]. Older age tended to increase the probability of more severe intracranial injuries. Other studies have reported that increasing age is related to poorer outcome after TBI [44]. Of all patients with severe intracranial injuries, 20% were young adults living with parents and attending secondary school. School education programmes about the effects and dangers associated with alcohol consumption and drug use are "a key component in preventing substance abuse in this population" [14].

This study has several important limitations that should be addressed. It is a selected cohort study describing a TBI population aged 16-55 years. The study assesses the severity of anatomical brain injury as shown on CT at a fixed point of time (within 24 hours of injury). The clinical course and outcome of TBI were not evaluated in this study. Our results should be interpreted with caution because no standard protocol to obtain blood samples and urine tests for substance exists (25% of patients were classified by clinical judgment), as well as the small number of participants. Therefore, we made no distinction between the types of substances used when analysing the probability of more severe TBI. The frequency of pre-injury substance problems is slightly underestimated due to the exclusion criteria which resulted in omission of 14 patients with previously diagnosed severe substance abuse. Ten of these suffered from severe TBI supporting the association between pre-injury substance abuse and severe structural brain injury shown by the study. Self-reported screening instruments might under-report pre-injury use of alcohol and, especially, of illicit drugs. Limitations of the CAGE questionnaire are described above. Clinical interviews based on the DSM-IV diagnostic criteria may provide a reliable measure of substance abuse disorders. However, a replication of the study is needed before the present findings can be considered as a verified hypothesis.

## Conclusion

One in two patients was under the influence on admission, and one in five abused substances pre-injury. Substance use on admission was more frequent in less severe TBI caused mostly by low-energy events such falls, violence and sport accidents. Pre-injury substance abuse increased the probability of more severe structural brain injuries. These were mostly results of high-energy events such as motor vehicle accidents and falls from higher levels. Targeting preventive efforts to reduce substance use and misuse in the TBI population in general is needed in order to minimise the number of injuries and consequences including the socio-economic burden. In view of the trend of aging in the general population and the differences in the mechanism of injuries between younger and older individuals, studies on the association between substance use and TBI in the elderly are also needed.

## Competing interests

The authors declare that they have no competing interests.

## Authors' contributions

NA and CR were responsible for the conception and design of the study. NA and SS collected data. TJ reviewed the CT findings and performed Marshall Classification. LS helped with statistical analysis and interpretation of data. SS and AKS participated in the study design. All authors contributed to the analysis and interpretation of data that was initially performed by NA. NA, SS and CR drafted the manuscript and all authors reviewed it critically for intellectual content and have given final approval of the version to be published.
